# Strategies for monitoring and updating clinical practice guidelines: a systematic review

**DOI:** 10.1186/1748-5908-7-109

**Published:** 2012-11-19

**Authors:** Laura Martínez García, Ingrid Arévalo-Rodríguez, Ivan Solà, R Brian Haynes, Per Olav Vandvik, Pablo Alonso-Coello

**Affiliations:** 1 Iberoamerican Cochrane Centre, Biomedical Research Institute Sant Pau (IIB Sant Pau), Barcelona, Spain; 2 Clinical Research Institute, GETS, Universidad Nacional de Colombia, Bogotá, Colombia; 3 McMaster University, Hamilton, Canada; 4 Norwegian Knowledge Centre for the Health Services, Oslo, Norway

**Keywords:** Clinical practice guidelines, Diffusion of innovation, Evidence-based medicine, Information storage and retrieval, Methodology, Updating, Implementation science, Dissemination and implementation, Knowledge translation

## Abstract

**Background:**

Scientific knowledge is in constant change. The flow of new information requires a frequent re-evaluation of the available research results. Clinical practice guidelines (CPGs) are not exempted from this phenomenon and need to be kept updated to maintain the validity of their recommendations. The objective of our review is to systematically identify, describe and assess strategies for monitoring and updating CPGs.

**Study design and setting:**

We conducted a systematic review of studies evaluating one or more methods of updating (with or without monitoring) CPGs or recommendations. We searched MEDLINE (PubMed) and The Cochrane Methodology Register (The Cochrane Library) from 1966 to June 2012. Additionally, we hand-searched reference lists of the included studies and the Guidelines International Network book of abstracts. If necessary, we contacted study authors to obtain additional information.

**Results:**

We included a total of eight studies. Four evaluated if CPGs were out of date, three updated CPGs, and one continuously monitored and updated CPGs. The most detailed reported phase of the process was the identification of new evidence. As opposed to studies updating guidelines, studies evaluating if CPGs were out of date applied restricted searches. Only one study compared a restricted versus an exhaustive search suggesting that a restricted search is sufficient to assess recommendations’ Validity. One study analyzed the survival time of CPGs and suggested that these should be reassessed every three years.

**Conclusions:**

There is limited evidence about the optimal strategies for monitoring and updating clinical practice guidelines. A restricted search is likely to be sufficient to monitor new evidence and assess the need to update, however, more information is needed about the timing and type of search. Only the exhaustive search strategy has been assessed for the update of CPGs. The development and evaluation of more efficient strategies is needed to improve the timeliness and reduce the burden of maintaining the validity of CPGs.

## Background

Scientific knowledge is in constant change, and new information requires frequent assessment to determine whether it changes the knowledge base [1]. A clinical practice guideline (CPG) may be considered out of date if it does not include all recent, valid, and relevant evidence or does not reflect current clinicians’ experience and patients’ values and preferences [2]. CPGs, hence, need to be updated regularly to remain valid.

Shekelle et al. evaluated the validity of a cohort of CPGs [3]. Survival analysis indicated that 90% of CPGs were still valid in 3.6 years, but 50% were out of date in 5.8 years [3]. Based on these results, most methodological handbooks for the development of CPGs propose three years as a reasonable time frame to update their guidelines [1,4].

In 2007, Moher et al. conducted a study about when and how to update systematic reviews [5]. Although not included in the objectives, the authors identified and described several methods for updating CPGs. In their conclusions the authors argue that the methodology for updating CPGs, as opposed to systematic reviews, is well established. Nevertheless, a recent international survey showed high variability and a lack of standardization in the CPGs updating processes [6].

A few studies have evaluated different strategies for the CPGs updating process [3,7,8], however, no systematic reviews have been conducted about this topic. We therefore undertook a systematic review of the studies that assessed strategies for monitoring and updating CPGs.

## Methods

### Information sources and search

We performed a search in June 2012 in MEDLINE (accessed through PubMed, from 1966 onwards) and The Cochrane Methodology Register (accessed through *The Cochrane Library*, Issue 6 2012). We included studies regardless of their language or publication status. The search strategy is available as supplementary data (Additional file 1). Additionally, we hand searched reference lists of the included studies and in the Guidelines International Network book of abstracts (available online from 2009 until 2011 in http://www.g-i-n.net/). If necessary we contacted study authors to obtain additional information. Two authors were in charge of performing all searches (IS, LMG).

### Eligibility criteria

**Figure 1:**
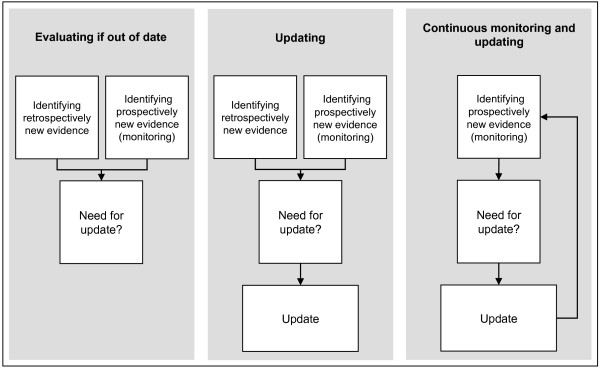
Updating Clinical Practice Guidelines Strategies.

1. Type of study: We included studies evaluating one or more methods of updating (with or without monitoring) evidence-based CPGs or recommendations. We excluded studies that only reported updating methods (without actually testing them), methodological handbooks or CPGs updates. We made no restriction by health topic.

2. Type of design: We included studies assessing strategies for evaluating if CPGs are out of date; for updating CPGs; for continuous monitoring and updating of CPGs (Figure 1).

### Study selection

Two authors independently reviewed the titles and abstracts, as well as the full text of the selected articles for a more detailed evaluation and approved their final inclusion (LMG, IAR). Any disagreement among the authors was initially resolved by consensus, and if necessary, we consulted a third author (IS).

### Data extraction strategy

Two authors independently extracted information from the included studies using an ad hoc form (LMG, IAR) that can be requested from the authors. Disagreements among the authors were resolved by consensus and, if required, by a third author (IS). We contacted study authors by email when more information was needed.

We extracted the following information from each study: institution or guideline program and country; objective and design of the study; sample (selection and size) and health topic; time to update (number of years since the development of the original CPG); stages of the strategies; type of search (restricted or exhaustive, classified depending on databases consulted and types of studies searched); resource use (number of participants and time spent); search and update results; and advantages and limitations of strategies reported by the authors.

### Data synthesis and presentation

We describe included studies both individually and narratively. We calculated the search performance of the strategies (as a proportion of included documents from all documents identified); and update performance of the strategies (as a proportion of updated recommendations or CPGs from all evaluated recommendations or CPGs).

## Results

### Study selection

**Figure 2:**
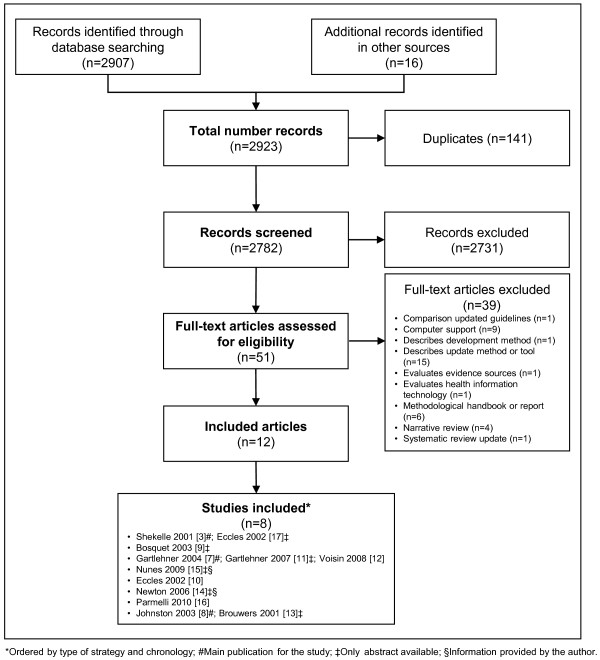
**Flow diagram for the identification of studies.** *Ordered by type of strategy and chronology; #Main publication for the study; ‡Only abstract available; §Information provided by the author.

The screening process is summarized in the flow diagram (Figure 2). We initially identified 2,923 references from our search strategy, and excluded 141 duplicates and 2,731 references after examining the title and abstract. We reviewed 51 full texts and we excluded 39 references (Additional file 2). We finally included eight studies corresponding to 12 original references [3,7-17]. Three studies were only available as abstracts [9,14,15]. We successfully contacted two of the first abstracts’ authors to obtain additional information of these studies [14,15].

### Study characteristics

Four studies assessed strategies evaluating if CPGs were out of date [3,7,9,15], three studies assessed strategies for updating CPGs [10,14,16], and one study assessed a strategy for continuous monitoring and updating CPGs [8] (Table 1). Five studies assessed a single strategy for: evaluating if CPGs were out of date [3,9,15], for updating CPGs [14] or for continuous monitoring and updating of CPGs [8]; three studies compared two strategies: two different strategies for evaluating if CPGs were out of date [7] or ‘de novo’ development versus updating strategies [10,16]. Most of the studies evaluated full CPGs (range from one to 20 CPGs) [3,8-10,14,15], however none of them included a random sample. The topics included varied widely, with cancer being the most frequent [8,9,16]. The time to update source documents ranged from one to eight years. The common stages identified in the strategies were: assessment of need to update; scope definition of the update; working group composition; search strategy; selection, assessment, and synthesis of the evidence; recommendations update (only for updating and continuous monitoring); and presentation format of the updates (for strategies for updating and continuous monitoring) (Table 2).

**Table 1:** Characteristics of included studies*

**Author and year**	**Institution or guideline program (country)**	**Objective**	**Design**	**Sample**	**Health topic**	**Time to update**
**Shekelle 2001**[3]	AHRQ (USA)	Evaluating if out of date	One strategy	17 CPGs	Several topics	4-8 years
**Bosquet 2003**[9]‡	FNCLCC (France)	Evaluating if out of date	One strategy	1 CPG	PET scanning in cancer	NS
**Gartlehner 2004**[7]	RTI-UNC EPC, USPSTF, AHRQ (USA)	Evaluating if out of date	Two strategies	6 topics	Prevention topics	6 years
**Nunes 2009**[15]‡§	NCGC, NICE (UK)	Evaluating if out of date	One strategy	1 CPG	Obesity	3 years
**Eccles 2002**[10]	North of England Evidence Based Guideline Development Programme (UK)	Updating	Two strategies (development vs updating)	2 CPGs	Angina and asthma in adults	4-5 years
**Newton 2006**[14]‡§	AHTA (Australia)	Updating (updating by adapting)	One strategy	1 CPG	Posttraumatic stress disorder	1-3 years
**Parmelli 2010**[16]	ERHCA (Italy)	Updating	Two strategies (development vs updating)	15 recommendations	Anticancer drugs for breast, colorectal and lung cancer	3 years
**Johnston 2003**[8]	CCOPGI (Canada)	Continuously monitoring and updating	One strategy	20 CPGs	Cancer	NS

*Ordered by type of strategy and chronology; ‡Only abstract available; §Information provided by the author.

Abbreviations: AHRQ: Agency for Healthcare Research and Quality; AHTA: Adelaide Health Technology Assessment; CCOPGI: Cancer Care Ontario Practice Guidelines Initative; CPG: Clinical practice guideline; ERHCA: Emilia-Romagna Health Care Agency; FNCLCC: Fédération Nationale des Centres de Lutte Contre le Cancer; NCGC: National Clinical Guidelines Centre for Acute and Chronic Conditions; NICE: National Institute for Health and Clinical Excellence; NS: Not stated; PET: Positron Emission Tomography; RTI-UNC EPC: RTI International–University of North Carolina Evidence-based Practice Center; UK: United Kindom; USA: United States of America; USPSTF: US Preventive Services Task Force; vs: Versus.

**Table 2:** Stages of the strategies*

**Author and year**	**Assessing the need to update**	**Scope definition**	**Update group (number of participants)**	**Evidence search strategy**	**Evidence selection, assessment and synthesis**	**Update recommendations**	**Presentation of updates**
**Shekelle 2001**[3]	Within specific timeframe	Identification of main recommendations	Reviewers (2) Chairs of the original CPG expert panel (15) Members related with original CPGs (2) Members of the original CPG expert panel (121) Nonpanel experts (8)	**Restricted search** 5 key medical journals and key specialty journals Reviews, editorials, commentaries Additional search in NGC and web sites of CPGs publishing organizations Surveyed experts by mail	Reviewed references (title, abstract and articles) Reviewed the relevance of selected references Based on new evidence and judgment guidelines were classified in to: major update, minor update or still valid	NA	NA
**Bosquet 2003**[9]‡	Monitoring	Discussion about priority of topics to be updated	Methodologist (NS) Original working group (NS)	**Exhaustive search** MEDLINE, EMBASE, SBU and APM web sites	Selection of references Evaluation the impact of reference on existing guideline: consistent with existing guidelines, inconsistent or concern new topics	NA	NA
**Gartlehner 2004**[7]	Within specific timeframe	Developed analytic framework, critical key questions and eligibility criteria Survey to national/ international experts	Clinicians (4) Health services researchers (2) Reviewers (4) Librarians (NS) National or international experts (13)	**Modified Shekelle et al. [Shekelle2001] search** AIM journals and non-AIM speciality journals Reviews, editorials, commentaries, guidelines Additional search in PreMedline, HSTAT, The Cochrane Library and selected NIH websites Surveyed to national or international experts **Exhaustive search** MEDLINE, The Cochrane Library Reviews, meta-analysis, RCTs Surveyed to national or international experts	Assessed the relevance of abstracts, full-text articles and original studies Used of update memos with studies that judged as eligible and addressed a critical key question	NA	NA
**Nunes 2009**[15]‡§**	Within specific timeframe	NS	Methodologist (3) Clinicians (3)	**Restricted search** Guidelines	Selection and assessment guidelines with AGREE Narrative summary of the methodological aspects and major findings of each guideline	NA	NA
**Eccles 2002**[10]	Within specific timeframe	NS	General practitioners (8) Consultants (2) Nurses (2) Health economists (2) Guideline methodologist (2)	**Exhaustive search** MEDLINE, EMBASE, The Cochrane Library	Systematic review Evidence tables or text summaries	3 situations: new recommendations; refinement recommendations; and unchanged recommendations	NS
**Newton 2006**[14]‡§	NS	Additional research questions	Methodologist (9) Clinicians (6)	**Exhaustive search** NS	Verification of results and study quality from the original review	Integrating a qualitative and quantitative data on the prior review and developing recommendations	NS
**Parmelli 2010**[16]	Within specific timeframe	Mail consultation to experts from development group Relevant evidence	Coordinating group (10) 3 multidisciplinary panels (NS) External methodological group (NS)	**Exhaustive search** MEDLINE, EMABASE, central and databases for conference (ASCO) SRs, meta-analysis, RCTs	Evidence selection Application GRADE methodology Evidence tables Informed and discussed with panellist in meetings	Classified strength of recommendation in 4 levels: strong positive, weak positive, weak negative and strong negative Voted by experts in meeting	NS
**Johnston 2003**[8]	Monitoring	NS	Health information specialist (3) Lead author of the original guideline (NS) DSG who developed guideline: chair, members and research assistant (NS)	**Exhaustive search** MEDLINE (monthly), CancerList (monthly), HealthStar (monthly), The Cochrane Library (quarterly) Guidelines, SRs, RCTs Additional search in key journals and proceedings meeting Notifications by DSG members	Identified potentially relevant new trials, meta-analyses an evidence-based guidelines or update results from trials included in original guideline Reviewed abstracts and articles Considered relevance of each reference to original guideline question Interpreted new evidence in the context of original guideline Descriptive and summaries of new evidence	DSG choose implications of new evidence on clinical recommendations: unchanged recommendation, strength recommendation, change recommendation	Notice linked to each guideline to keep practitioners aware that regular update search conduced Update bulletin with new evidence emerged Notice at the top of the guideline to alert practitioners that guideline was review Removed obsolete guidelines Integrated evidence update into original report (dynamic ‘living’ practice guidelines)

*Ordered by type of strategy and chronology; ‡Only abstract available; §Information provided by the author.

Abbreviations: *AIM* Abridged Index Medicus, *APM* Agence de Presse Médicale, *ASCO* American Society of Clinical Oncology, *CPG* Clinical practice guideline, *DSG* Disease site group, *GRADE* Grading of Recommendations Assessment, Development and Evaluation, *HSTAT* Health Service/Technology Assessment Text, *NGC* National Guideline Clearinghouse, *NIH* National Institutes of Health, *NA* Not applicable, *NS* Not stated, *RCT*. Randomized controlled trials, *SBU* Swedish Council on Health Technology Assessment, *SR* Systematic review.

### Strategies for evaluating if CPGs are out of date

Shekelle et al. [3] developed a strategy to assess the validity of CPGs based on identified new evidence through restricted searches (reviews, editorials, or commentaries in general or specialised journals) and through a survey of clinical experts (Table 2). The CPGs were classified by the type of update required as: major update—new evidence suggests the need for new recommendations; minor update—new evidence supports changes to recommendations or refinement of existing recommendations; and the recommendations remain valid. The participants in this updating process were two methodologists and 146 clinical experts. The clinical experts participated in a survey to assess current validity of the guideline recommendations and to identify new evidence (71% of response rate). The search performance was 2.9% (208 articles reviewed from 7,150 articles initially identified) (Table 3). The authors concluded that 76.5% of the guidelines needed to be updated (13/17 CPGs) (Table 3). The results of survival analysis indicated that 90% of CPGs were still valid at 3.6 years (95% confidence interval (CI) 2.6 to 4.6 years), but 50% of CPGs were out of date at 5.8 years (95% CI 5.0 to 6.6 years). The main reported limitation of the study included no previous validation of the method of assessing obsolescence.

**Table 3:** Results of the strategies*

**Author and year**	**Search performance (%)**	**Update performance (%)**
**Shekelle2001**[3]	208 articles reviewed/7150 articles initially identified (2,9)	13/17 CPGs (76,5)
**Bosquet 2003**[9]‡	118 references submitted to the working group/261 references initially identified (45,2)	NS
**Gartlehner 2004**[7]	Modified Shekelle et al. search: 36 eligible studies/1382 citations initially identified (2,6)	NS
	Exhaustive search: 45 eligible studies/3687 citations initially identified (1,2)	NS
**Nunes 2009**[15]‡§	7 guidelines reviewed/25 guidelines initially identified (28)	0/NS recommendations
**Eccles 2002**[10]	Angina CPG: 59 acceptable paper/5941 citations initially identified (1)	0/NS recommendations
	Asthma CPG: 79 acceptable paper/7560 citations initially identified (1)	0/NS recommendations
**Newton 2006**[14]‡§	43 studies included/19423 citations initially identified	11/NS questions
**Parmelli 2010**[16]	24 papers included/686 records initially screened (3,5)	6/15 recommendations (40)
**Johnston 2003**[8]	19 citations with impact on recommendation/80 citations initially identified (23,8)	6/20 CPGs (30)

*Ordered by type of strategy and chronology; ‡Only abstract available; §Information provided by the author.

Abbreviations: *CPG* Clinical practice guideline, *NS* Not stated.

Bosquet et al. [9] monitored the literature to assess the need for updating a CPG (Table 2). This approach applied an exhaustive search and evaluated the impact of new studies on existing guidelines (consistent, inconsistent or concern new topics) by consultation with the original working group (mail and meetings). The search performance was 45.2% (118 references submitted to the working group from 261 references initially identified) (Table 3).

Gartlehner et al. [7] compared two search strategies to identify new evidence for assessing the need to update six topics of one CPG: a modified Shekelle et al. [3] search versus an exhaustive search (Table 2). The researchers modified in three consecutive phases the literature search developed by Shekelle et al. [3], mainly by eliminating some of the databases (omitting a general web site search and confining the MEDLINE search to Abridged Index Medicus journals). The participants were 10 methodologists (five for each model) and 13 clinical experts. The clinical experts participated in a survey similar to the one carried out in Shekelle et al. [3] (28% response rate). The search performance was 2.6% for the modified Shekelle et al. [3] search (36 eligible studies from 1,382 citations initially identified) and 1.2% for the exhaustive search (45 eligible studies from 3,687 citations initially identified) (Table 3). The reported strength of the modified Shekelle et al. [3] method was that it offered a search strategy that detected fewer citations to review and identified all studies that triggered an update. However, the study only included prevention topics; the comparison of the number of abstracts and full texts between the two approaches was limited because the Shekelle et al. [3] search was an integral part of the exhaustive search; and the experts who assessed the importance of the studies not identified were not blinded to the type of search approach.

Nunes et al. [15] described a restricted search and a review of new CPGs to decide whether their guideline required an update (Table 2). The participants were three methodologists and two experts. The search performance was 28% (seven guidelines reviewed from 25 guidelines initially identified) (Table 3). No recommendations needed to be updated because identified recommendations in other CPGs were consistent with the existing CPG (Table 3). Less time was required to perform the update strategy than the time estimated to perform an exhaustive search. The need to judge the applicability of recommendations from other guidelines was one of the difficulties reported.

### Strategies for updating CPGs

Eccles et al. [10] compared the updating process with the original development process of two CPGs (Table 2). In both the development and updating process new evidence was identified by exhaustive search. Recommendations were classified as: new (if new evidence was identified); refined (if supplementary evidence was identified); and unchanged (if no new evidence was identified). Two methodologists and 14 clinical experts participated. The search performance was 1.0% for each guideline (e.g., for angina there were 59 acceptable papers from 5,941 citations initially identified) (Table 3). Recommendations remained unchanged (Table 3). Among the reported strengths were that some members also had participated in the original development of the guidelines and were better trained and were more familiar with the literature. These members also identified other relevant evidence. The main reported limitation of the updating strategy was that it was as burdensome, time consuming, and costly.

Newton et al. [14] updated an existing CPG (by updating and expanding the original search) and adapted it to the local context (by adding research questions for the local setting) (Table 2). The participants were nine methodologists and six experts. The search performance was 0.2% (43 studies included from 19,423 citations initially identified) (Table 3). The authors spent nine months to update 11 research questions and to develop seven new research questions (Table 3). The authors acknowledged that they were uncertain whether the process was more time efficient due to the additional search needed to cover local adaptation issues.

Parmelli et al. [16], similarly to Eccles et al. [10], compared an updating method with the original development process in a set of 15 recommendations (Table 2). The GRADE framework was used in both developing and updating recommendations [18]. For the updating process, new exhaustive searches were designed. The number of participants was not specified, but they described the main characteristics of its coordinator, methodological, and expert group. The researchers spent eight months to update 15 recommendations. The search performance was 3.5% (24 papers included from 686 records initially screened) (Table 3). Forty per cent of the recommendations (6/15) were completely updated due to identification of new evidence, leading to a change in recommendation classification or to redefinition of the original clinical questions (Table 3). The advantages of this updating strategy, as reported by the authors, were better collaboration among the members of the updating group, and improved methodology, leading to more relevant recommendations for clinical practice. On the other hand, the updating process was neither time nor resource saving.

### Strategies for continuous monitoring and updating of CPGs

Johnston et al. [8] carried out a pilot study of a monitoring strategy in 20 CPGs (Table 2). The strategy included four steps: continuous and exhaustive searching; reviewing the new evidence; revising recommendations; and announcing new evidence and modified recommendations. The researchers used the term ‘living’ practice guideline, defined as ‘integrated evidence update into the original report.’ The information about the participants was limited. The researchers applied this strategy during a year. The search performance was 23.75% (19 citations with impact on recommendations from 80 citations initially identified) (Table 3). In 30% (6/20) of the guidelines, a change in their clinical recommendations was made (Table 3). The reported advantages of this method were that often the experts identified new evidence before it was available in electronic databases, and they therefore proposed that the literature search could be done quarterly and limited to Medline, the Cochrane library, and meeting proceedings. The authors described the process as intensive, and they foresaw that the updating process would need to change if many more guidelines had to be updated.

## Discussion

Our systematic review shows that the available research about the monitoring and updating of CPGs is scarce, and little is known at present about the most efficient way of keeping guidelines valid. Furthermore, the suboptimal reporting and the wide variability in the design, choice of outcomes, type of search strategy, and breadth of topics makes the assessment of the included studies difficult.

We identified eight studies with three main different objectives: evaluation if guidelines are out of date and, hence, how often they need updating [3,7,9,15], and the evaluation of updating strategies without [10,14,16] or with continuous monitoring new evidence [8]. Regardless of the goal, the included studies followed similar stages in their process. However, authors generally did not describe the process in enough detail to clearly identify the different stages.

The most detailed phase of the studies was the literature search method. Strategies evaluating if CPGs were out of date [3,7] used more restricted searches (limited to reviews, editorials or commentaries of specific journals and expert collaboration) than updating strategies. These approaches aim to identify the relevant new evidence without performing an exhaustive search; however, they risk omitting key references (references that trigger a modification of a recommendation). The evidence for their performance is so far limited [3,7], with only one single head-to-head comparison available between a restricted search and an exhaustive search [7]. According to the results observed in Gartlehner et al. [7], the evidence identified by this more restricted search would be enough to assess the need to update a CPG. However, it remains unclear whether these search results would be a sufficient way to actually update recommendations.

Strategies that aimed to update [10,16] or continuously monitor and update CPGs [8] used more exhaustive searches and were generally very similar to the ones used in the development processes. These exhaustive search strategies are more time-consuming than restricted searches as the searches trade what is hoped will be higher sensitivity for low specificity. However, the study of Gartlehner et al. [7] suggests that sensitivity for key studies is not increased by an exhaustive search process.

Although search strategies were specified in the included studies, search results were difficult to compare across studies due to the variability of the search performance outcomes (e.g., Shekelle*et al.*[3] reported the articles reviewed and Gartlehner et al. [7] reported just the eligible studies). Furthermore, reported results would need to be adjusted by the number of CPGs or recommendations updated and the time to update them. Unfortunately, this information was not available for most of the studies.

Only three studies [3,8,16] reported updating performance results. For example, Parmelli et al. updated 40% of the recommendations about breast, colorectal, and lung cancer treatment three years after the development of the recommendations [16]. The update process should focus on the recommendations, instead of the full guideline, because it could provide an opportunity to make the process more efficient. The GRADE system [19], already used by Parmelli et al. [16], could provide a framework to systematically and explicitly assess to what extent the new evidence can modify the different factors that ultimately influence the direction and strength of the recommendations (quality of the evidence, balance between benefits and harms, patients’ values and preferences, and resource use) [18].

Future research studies should standardize outcomes of interest. In relation to the search performance, authors should report the number of key references (references that triggered a modification of a recommendation) from the number of references initially identified. In relation to the updating performance, measures should include the number of recommendations updated from total of the CPG recommendations. Finally, regarding resource use, studies should report the number of participants and the time spent in the updating process.

All the studies described the composition of the updating working group involved in the process; however, the total number of participants and their roles was generally unclear. In general, similarly to the development of guidelines, there was a core method group and a larger group of clinical experts (with a variable degree of involvement). Only three studies included the time devoted to the process (range eight to ten months) [8,14,16]. Nevertheless, this information is difficult to generalise because it is highly dependent on the goal of the strategy, the scope of the guideline, the methodological expertise of the group members or the economic resources of the different institutions involved, among other possible factors. Had this information been more detailed, it could have been used as a guide to assist in the development and updating of CPGs programs.

There is a need to develop more efficient monitoring and updating strategies for CPGs and, for this, rigorous research is crucial. The gold standard strategy to update CPGs should include the identification of new evidence by an exhaustive search and the update of the recommendations [10,16]. One of the most resource-intensive phases where there is more room for improvement is the literature search. Other restricted search strategies or resources like McMaster Premium LiteratUre Service or Clinical Queries, have shown to improve the efficacy of keeping systematic reviews up to date [20]. This remains to be replicated in guidelines. Other areas to explore are the performance of databases (e.g., EMBASE) or the role of clinical experts as a source of references, with these more limited strategies [21].

In relation to survival time, Shekelle et al. [3] proposed, as a general rule, to assess the validity of CPGs every three years. Regardless of the time frame, which is highly dependent on the health topic, it would be desirable to develop a dynamic warning system to identify new relevant evidence (monitoring) and evaluate the need to update. A potential tool that could be easily implemented to monitor the new evidence is the automated periodic searches in MEDLINE, using their Selective Dissemination of Information service [22]. Complementary, guideline groups could monitor publication rate of specific MeSH terms in relation with a CPG topic. At the moment this information is not readily available in biomedical databases (e.g., MEDLINE).

Our study has several strengths. We performed an exhaustive systematic review including contacting authors for additional information. In our review, we proposed a novel classification of CPGs updating strategies according to their objective: evaluation CPGs obsolescence, updating, and continuously monitoring and updating CPGs. Finally, we also propose standardised reporting framework, including outcomes, for research purposes.

Our study has limitations. First, it is possible that we did not identify all studies due to publication bias or to the omission of some more specialised information sources (e.g., conference proceedings). Second, the difficulty of synthesizing, evaluating, and comparing complex methodological studies, without a standardized reporting, as opposed to systematic reviews [23] or comparative effectiveness reviews [24], makes the analysis and interpretation of results challenging.

## Conclusions

There is limited evidence about the optimal strategies for monitoring and updating CPGs. A restricted search is likely to be sufficient to monitor new evidence and assess the need to update; however, more information is needed about the timing and type of search. Only the exhaustive search strategy has been assessed for the update of CPGs.

Our review highlights suboptimal reporting, and wide variability in the design, choice of outcomes, and type of search strategy of the available studies. The development and evaluation of more efficient strategies is needed to improve the timeliness and reduce the burden of maintaining the validity of CPGs. Future research studies should adequately report their methods and results.

## Abbreviations

CI: Confidence interval; CPG: Clinical practice guideline.

## Competing interests

The authors declare that they have no competing interests.

## Authors’ contributions

Conceiving the review: LMG, PAC. Undertaking searches: IS, LMG. Screening search results: LMG, Ingrid IAR. Screening retrieved papers against inclusion criteria: LMG, IAR, IS. Extracting data from papers: LMG, IAR. Data management for the review: LMG. Writing the review: LMG, PAC. Comment and editing of review drafts: LMG, PAC, IAR, IS, R Brian Haynes, Per Olav Vandvik, Petra Díaz del Campo Fontecha, Maria Dolors Estrada Sabadell, Elvira García Álvarez, Javier Gracia San Román, Anna Mariyanova Kotzeva, Flavia Salcedo Fernandez, María del Mar Trujillo Martín. Responsible for reading and checking review before submission: LMG, PAC. All authors read and approved the final manuscript.

## Supplementary Material

Additional file 1 **Search strategy.** This document shows the search strategy in MEDLINE and The Cochrane Methodology Register. Click here for file

Additional file 2 **Excluded studies.** This document shows a list with excluded studies and the reason for exclusion. Click here for file

## References

[B1] Working Group on CPG Updates Updating Clinical Practice Guidelines in the Spanish National Health System: Methodology Handbook 2009 Madrid: National Plan for the National Health System of the Spanish Ministry for Health and Social Policy; Aragon Health Sciences Institute (I+CS); Clinical Practice Guidelines in the Spanish National Health System: I+CS

[B2] Clark E Donovan EF Schoettker P From outdated to updated, keeping clinical guidelines valid Int J Qual Health Care 2006 18 3 165 166 10.1093/intqhc/mzl007 16613986

[B3] Shekelle PG Ortiz E Rhodes S Validity of the Agency for Healthcare Research and Quality clinical practice guidelines: how quickly do guidelines become outdated? JAMA 2001 286 12 1461 1467 10.1001/jama.286.12.1461 11572738

[B4] National Institute for Health and Clinical Excellence (January 2009) The guidelines manual 2009 London: National Institute for Health and Clinical Excellence Available from: http://www.nice.org.uk.

[B5] Moher D Tsertsvadze A Tricco AC Eccles M Grimshaw J Sampson M A systematic review identified few methods and strategies describing when and how to update systematic reviews J Clin Epidemiol 2007 60 11 1095 1104 17938050 10.1016/j.jclinepi.2007.03.008

[B6] Alonso-Coello P Martínez García L Carrasco JM Solà I Qureshi S Burgers JS The updating of clinical practice guidelines: insights from an international survey Implement Sci 2011 6 107 10.1186/1748-5908-6-107 21914177 PMC3191352

[B7] Gartlehner G West SL Lohr KN Assessing the need to update prevention guidelines: a comparison of two methods Int J Qual Health Care 2004 16 5 399 406 10.1093/intqhc/mzh081 15375101

[B8] Johnston ME Brouwers MC Browman GP Keeping cancer guidelines current: results of a comprehensive prospective literature monitoring strategy for twenty clinical practice guidelines Int J Technol Assess Health Care 2003 19 4 646 655 15095770 10.1017/s0266462303000606

[B9] Bosquet L Guillo S Gory-Delabaere G Fervers B COSOR Technological and scientific literature monitoring for updating clinical practice guidelines: example with use of PET scanning in patients with cancer Improving Outcomes Through Health Technology Assessment. 19th Annual Meeting of the International Society of Technology Assessment in Health Care 2003 Alberta, Canada: Canmore 272

[B10] Eccles M Rousseau N Freemantle N Updating evidence-based clinical guidelines J Health Serv Res Policy 2002 7 2 98 103 10.1258/1355819021927746 11934374

[B11] Gartlehner G West SL Lohr KN Assessing the need to update prevention guidelines: a comparison of two methods Evid Base Libr Inform Pract 2007 2 2 40 41 10.1093/intqhc/mzh081 15375101

[B12] Voisin CE de la Varre C Whitener L Gartlehner G Strategies in assessing the need for updating evidence-based guidelines for six clinical topics: an exploration of two search methodologies Health Info Libr J 2008 25 3 198 207 10.1111/j.1471-1842.2007.00765.x 18796080

[B13] Brouwers M Johnston M Browman G Results of a prospective study to keep guidelines current17th Annual Meeting of the International Society of Technology Assessment in Health Care: Building Bridges Between Policy, Providers, Patients and Industry 2001 Philadelphia, Pennsylvania, USA

[B14] Newton S Merlin T Forbes D Phelps A Creamer M Hiller J Challenges in updating evidence-based clinical practice guidelines Third Annual Meeting Health Technology Assessment International 2006 Adelaide, Australia 141

[B15] Nunes V Shaw E When to Update Guidelines. A pragmatic approach Proceedings of the 6th Guidelines International Network Conference 2009 Lisbon

[B16] Parmelli E Papini D Moja L Bandieri E Belfiglio M Ciccone G Updating clinical recommendations for breast, colorectal and lung cancer treatments: an opportunity to improve methodology and clinical relevance Ann Oncol 2011 22 1 188 194 10.1093/annonc/mdq324 20605933

[B17] Eccles M Shekelle P Grimshaw J Woolf S Updating of CPGs. Experiences from the U.K. (& USA). The Challenge of Collaboration Satellite Symposium Clinical Practice Guidelines 2002 Berlin, Germany: International Society of Technology Assessment in Health Care 238

[B18] Guyatt GH Oxman AD Vist GE Kunz R Falck-Ytter Y Alonso-Coello P GRADE: an emerging consensus on rating quality of evidence and strength of recommendations BMJ 2008 336 7650 924 926 10.1136/bmj.39489.470347.AD 18436948 PMC2335261

[B19] Guyatt GH Oxman AD Schünemann HJ Tugwell P Knottnerus A GRADE guidelines: a new series of articles in the Journal of Clinical Epidemiology J Clin Epidemiol 2011 64 4 380 382 10.1016/j.jclinepi.2010.09.011 21185693

[B20] Hemens BJ Haynes RB McMaster Premium LiteratUre Service (PLUS) performed well for identifying new studies for updated Cochrane reviews J Clin Epidemiol 2012 65 1 62 72 10.1016/j.jclinepi.2011.02.010 21856121

[B21] Shekelle PG Newberry SJ Wu H Suttorp M Motala A Lim Y-W Identifying Signals for Updating Systematic Reviews: A Comparison of Two Methods Methods Research Report. AHRQ Publication No. 11-EHC042-EF 2011 Rockville (MD): Agency for Healthcare Research and Quality Available at http://effectivehealthcare.ahrq.gov/. 21834176

[B22] Shultz M De Groote SL MEDLINE SDI services: how do they compare? J Med Libr Assoc 2003 91 4 460 467 14566377 PMC209512

[B23] Moher D Tsertsvadze A Tricco A Eccles M Grimshaw J Sampson M When and how to update systematic reviews Cochrane Database of Systematic Reviews 2008, Issue 1. Art. No.: MR000023 2008 10.1002/14651858.MR000023.pub3 PMC8941847 18254126

[B24] Tsertsvadze A Maglione M Chou R Garritty C Coleman C Lux L Updating Comparative Effectiveness Reviews: Current Efforts in AHRQ’s Effective Health Care Program. Methods Guide for Comparative Effectiveness Reviews (Prepared by the University of Ottawa EPC, RAND Corporation–Southern California EPC, Oregon EPC, University of Connecticut EPC, RTI–University of North Carolina EPC, Johns Hopkins Bloomberg School of Public Health EPC under Contract No. 290-02-0021 EPC2). AHRQ Publication No. 11-EHC057-EF 2011 Rockville, MD: Agency for Healthcare Research and Quality Available at: http://www.effectivehealthcare.ahrq.gov/reports/final.cfm.

