# Supplementary material for: Strategies for monitoring and updating clinical practice guidelines: a systematic review
Source: Implement Sci. 2012 Nov 19;7:109. doi: 10.1186/1748-5908-7-109 (PMC3520818; doi:10.1186/1748-5908-7-109)
Supplement: Additional file 1 — Search strategy. This document shows the search strategy in MEDLINE and The Cochrane Methodology Register. [file 1748-5908-7-109-S1.doc]

### Additional file 1: Search strategy (June 15^th^ 2012)

MEDLINE (PubMed)

#1 Clinical Practice Guideline*[tw] 5830

#2 Clinical guideline*[tiab] 5509

#3 Guideline*[ti] 42590

#4 Updat*[tw] 69510

#5 Up to date[tw] 9969

#6 (#1 OR #2) OR #3 48302

#7 #4 OR #5 78504

#8 #6 AND #7 **2781**

The Cochrane Methodology Register (*The Cochrane Library*)

#1 clinical practice guideline* 7963

#2 clinical guideline* 11911

#3 guideline*:ti 1568

#4 (#1 OR #2 OR #3) 12425

#5 updat* 374634

#6 up to date 105931

#7 (#5 OR #6) 404782

#8 (#4 AND #7) 10503

There are **126** results out of 15388 records for: "(#4 AND #7) in Cochrane Methodology Register"

**Tags used in PubMed search syntax**

- **[ti]:** Words and numbers included in the title of a citation.
- **[tiab]:** Words and numbers included in the title, abstract, and other abstract of a citation.
- **[tw]:** Includes all words and numbers in the title, abstract, other abstract, MeSH terms, MeSH Subheadings, Publication Types, Substance Names, Personal Name as Subject, Corporate Author, Secondary Source, and Other Terms (see Other Term [OT] above) typically non-MeSH subject terms (keywords), including NASA Space Flight Mission, assigned by an organization other than NLM.
