# Supplementary material for: Strategies for monitoring and updating clinical practice guidelines: a systematic review
Source: Implement Sci. 2012 Nov 19;7:109. doi: 10.1186/1748-5908-7-109 (PMC3520818; doi:10.1186/1748-5908-7-109)
Supplement: Additional file 2 — Excluded studies. This document shows a list with excluded studies and the reason for exclusion. [file 1748-5908-7-109-S2.doc]

### Additional file 2: Excluded studies [ordered by first author]

|  | **Reference** | **Reason for exclusion** |
| --- | --- | --- |
| 1 | Process for determining need for updates of clinical practice guidelines--AHCPR. Fed Regist. 1994;59(79 Pt 1):19723-5. | Methodological handbook or report |
| 2 | Clinical guidelines are not immortal: when do clinical guidelines become outdated? How can you know when an update is necessary? Prescrire Int. 2003;12(68):233-4. | Narrative review |
| 3 | Boer MM, Kersten S. Fast track guideline update successful. Proceedings of the 8^th^ Guidelines International Network Conference; 2011 Aug 28-31; Seoul, Korea. p. 106-7. | Describes update method or tool |
| 4 | Bosch M, Tavender E, Bragge P, Gruen R, Green S. How to define 'best practice' for use in Knowledge Translation research: a practical, stepped and interactive process. J Eval Clin Pract. 2012 Apr 9. | Methodological handbook or report |
| 5 | Bouaud J, Seroussi B. Characterizing the dimensions of clinical practice guideline evolution. Stud Health Technol Inform. 2008;136:139-44. | Computer support |
| 6 | Brouwers MC, Rawski E, Spithoff K, Oliver TK. Inventory of Cancer Guidelines: a tool to advance the guideline enterprise and improve the uptake of evidence. Expert Rev Pharmacoecon Outcomes Res. 2011;11(2):151-61. | Describes update method or tool |
| 7 | Clark E, Donovan EF, Schoettker P. From outdated to updated, keeping clinical guidelines valid. Int J Qual Health Care. 2006;18(3):165-6. | Narrative review |
| 8 | De Palma R, Liberati A, Ciccone G, Bandieri E, Belfiglio M, Ceccarelli M,et al. Developing clinical recommendations for breast, colorectal, and lung cancer adjuvant treatments using the GRADE system: a study from the Programma Ricerca e Innovazione Emilia Romagna Oncology Research Group. J Clin Oncol. 2008;26(7):1033-9. | Describes development method |
| 9 | Fishman L, Weinbrenner S, Ollenschläger G. Analysis of chronic respiratory disease guideline updates of the past 10 years. Proceedings of the 7^th^ Guidelines International Network Conference; 2010 Aug 25–28; Chicago, Illinois USA. p. 53. | Comparison updated guidelines |
| 10 | Gillis AM, Skanes AC. Canadian Cardiovascular Society Atrial Fibrillation Guidelines 2010: Implementing GRADE and Achieving Consensus. Can J Cardiol 2011; 27(1):27-30. | Describes update method or tool |
| 11 | Glover S. Guidance review: Issues, methods, and the role of the Information Specialist. Proceedings of the 7^th^ Guidelines International Network Conference; 2010 Aug 25–28; Chicago, Illinois USA. p. 101. | Narrative review |
| 12 | Guirguis-Blake J, Calonge N, Miller T, Siu A, Teutsch S, Whitlock E; U.S. Preventive Services Task Force.Current processes of the U.S. Preventive Services Task Force: refining evidence-based recommendation development.Ann Intern Med. 2007;147(2):117-22. | Methodological handbook or report |
| 13 | Honkanen M, Sipilä R, Komulainen J, Ketola E. Improving the updating process of current care guidelines. Proceedings of the 7^th^ Guidelines International Network Conference; 2010 Aug 25–28; Chicago, Illinois USA. p. 101-2. | Describes update method or tool |
| 14 | Kaiser K, Miksch S. Versioning computer-interpretable guidelines: semi-automatic modeling of 'Living Guidelines' using an information extraction method. Artif Intell Med. 2009;46(1):55-66. | Computer support |
| 15 | Kumar A, Quaglini S, Stefanelli M, Ciccarese P, Caffi E. Modular representation of the guideline text: an approach for maintaining and updating the content of medical education. Med Inform Internet Med. 2003;28(2):99-115. | Computer support |
| 16 | Latchem S, Alderson P. The process and outcomes of reviews of the need to update NICE guidelines. Proceedings of the 7^th^ Guidelines International Network Conference; 2010 Aug 25–28; Chicago, Illinois USA. p. 53-4. | Describes update method or tool |
| 17 | Latoszek-Berendsen A, Tange H, van den Herik HJ, Hasman A. From clinical practice guidelines to computer-interpretable guidelines. A literature overview. Methods Inf Med 2010; 49(6):550-70. | Computer support |
| 18 | MacDougall C , Percival J, McGregor C. Integrating health information technology into clinical guidelines. Conf Proc IEEE Eng Med Biol Soc. 2009:4646-9. | Evaluates health information technology |
| 19 | Moher D, Tsertsvadze A, Tricco AC et al. A systematic review identified few methods and strategies describing when and how to update systematic reviews. J Clin Epidemiol. 2007;60(11):1095-104. | Systematic review update |
| 20 | National Institute for Clinical Excellence (February 2004, updated 2005) Guideline Development Methods: Information for National Collaborating Centres and Guideline Developers. London: National Institute for Clinical Excellence. Available from: www.nice.org | Methodological handbook or report |
| 21 | Olver I, von Dincklage J. Use of wiki technology to develop and update cancer care guidelines. Proceedings of the 8^th^ Guidelines International Network Conference; 2011 Aug 28-31; Seoul, Korea. p. 61. | Describes update method or tool |
| 22 | Oxman AD, Schunemann HJ, Fretheim A. Improving the use of research evidence in guideline development: 16. Evaluation . Health Res Policy Syst. 2006;4:28. | Narrative review |
| 23 | Parkhill A, Hill K. Identifying the effective evidence sources to use in developing Clinical Guidelines for Acute Stroke Management: lived experiences of the search specialist and project manager. Health Info Libr J. 2009;26(1):47-55. | Evaluates evidence sources |
| 24 | Pau AK, Dutcher G, Cadden C, Nochetto F. Electronic dissemination of US HIV Treatment Guideline as a living document: Free access for a global audience.  Proceedings of the 7^th^ Guidelines International Network Conference; 2010 Aug 25–28; Chicago, Illinois USA. p. 56. | Describes update method or tool |
| 25 | Peleg M, Kantor R. Approaches for guideline versioning using GLIF. AMIA Annu Symp Proc. 2003;509-13. | Computer support |
| 27 | Qaseem A, Snow V, Owens DK, Shekelle P; Clinical Guidelines Committee of the American College of Physicians.The development of clinical practice guidelines and guidance statements of the American College of Physicians: summary of methods. Ann Intern Med. 2010;153(3):194-9. | Methodological handbook or report |
| 26 | Qaseem A, Forland F, Macbeth F, Ollenschläger G, Phillips S, van der Wees P; Board of Trustees of the Guidelines International Network. Guidelines International Network: toward international standards for clinical practice guidelines. Ann Intern Med. 2012 Apr 3;156(7):525-31. | Methodological handbook or report |
| 28 | Rios M, Desandes E, Bresson B et al. [Clinical practice guidelines in cancerology: comparative study of three decision support-systems for breast and prostate cancer in Lorraine french region]. Bull Cancer. 2003;90(4):363-70. | Computer support |
| 29 | Scott A, Moga C, Taenzer P, Findlay T, Harstall C. Updating adapted guidelines: How to streamline the process without losing rigour. Proceedings of the 8^th^ Guidelines International Network Conference; 2011 Aug 28-31; Seoul, Korea. p. 33. | Describes update method or tool |
| 30 | Scott-Wright AO, Fischer RP, Denekamp Y, Boxwala AA. A methodology for modular representation of guidelines. Stud Health Technol Inform. 2004;107(Pt 1):149-53. | Computer support |
| 31 | Serban R, ten Teije A, van Harmelen F, Marcos M, Polo-Conde C. Extraction and use of linguistic patterns for modelling medical guidelines. Artif Intell Med. 2007;39(2):137-49. | Computer support |
| 32 | Seyfang A, Martinez-Salvador B, Serban R, Wittenberg J, Miksch S, Marcos M. Maintaining formal models of living guidelines efficiently. In: Bellazzi R, Abu-Hanna A, Hunter J editors. Proceedings of the 11th conference on Artificial Intelligence in Medicine (AIME’07), LNAI 4594; 2007; Amsterdam, NL: Springer Verlag; p. 441-5. | Computer support |
| 33 | Shekelle P, Eccles MP, Grimshaw JM, Woolf SH. When should clinical guidelines be updated? BMJ. 2001;323(7305):155-7. | Describes update method or tool |
| 34 | Sparrow K, Lavibond K, Rabar S. A rapid update to a guideline: when new evidence questions the safety of a recommendation. Proceedings of the 8^th^ Guidelines International Network Conference; 2011 Aug 28-31; Seoul, Korea. p. 32. | Describes update method or tool |
| 35 | Theobald S, Blanc-Vincent MP, Farsi F et al. The identification of questions in the updating process of clinical practice guidelines for oncology [abstract]. 15th Annual Meeting of the International Society of Technology Assessment in Health Care; 1999 Jun 20-23; Edinburgh, UK 1999; 90. | Describes update method or tool |
| 36 | Washington DL, Bernstein SJ, Kahan JP, Leape LL, Kamberg CJ, Shekelle PG. Reliability of clinical guideline development using mail-only versus in-person expert panels. Med Care. 2003;41(12):1374-81. | Describes update method or tool |
| 37 | Weissbach L. [Which components should living guidelines contain?]. Urologe A. 2012;51(1):57-9. | Describes update method or tool |
| 38 | Zarnke KB, Campbell NR, McAlister FA, Levine M; Canadian Hypertension Recommendations Working Group.A novel process for updating recommendations for managing hypertension: rationale and methods.Can J Cardiol. 2000;16(9):1094-102. | Describes update method or tool |
| 39 | Zelman Lewis S. Sustainable Living Guidelines: A Model for the Future. Proceedings of the 8^th^ Guidelines International Network Conference; 2011 Aug 28-31; Seoul, Korea. p. 30. | Describes update method or tool |
